# Bisphenol A, 4-*tert*-Octylphenol, and 4-Nonylphenol in The Gulf of Gdańsk (Southern Baltic)

**DOI:** 10.1007/s00244-014-0023-9

**Published:** 2014-04-22

**Authors:** Marta Staniszewska, Lucyna Falkowska, Paweł Grabowski, Justyna Kwaśniak, Stella Mudrak-Cegiołka, Andrzej R. Reindl, Adam Sokołowski, Emilia Szumiło, Aleksandra Zgrundo

**Affiliations:** Institute of Oceanography, University of Gdańsk, Al. Marszałka Piłsudskiego 46, 81-378 Gdynia, Poland

## Abstract

The organic derivatives of phenol are classed as dangerous compounds, and their presence has been detected in surface water, bottom water, phytoplankton, zooplankton, and mussel as well as liver and muscle of fish from the Gulf of Gdańsk and in liver, muscle, and guano of gulls residing in the coastal zone of this basin. The greatest sources of bisphenol A (BPA), 4-*tert*-octylphenol (OP), and 4-nonylphenol (NP) were found to be the Vistula River and the water purification plant in Dębogórze. In living organisms, concentrations of BPA, OP, and NP ranged between the limit of quantification and several hundred ng g^−1^ dry weight (dw), and the highest concentrations were found for BPA. Prolonged alimentary exposure to BPA, OP, and NP in fish and birds was indicated by liver/muscle concentration ratios generally >1. The most influential factors on BPA and alkylphenol concentrations in the tissues of fishes and gulls were mainly diet and habitat. The study confirmed possible bioaccumulation in the food web. High BPA and NP concentrations in guano (≤2,700 and ≤300 ng g^−1^ dw, respectively) indicated the ability of birds to detoxify and signalled the reintroduction of these compounds to seawater. Herring, flounder, and cod from the Southern Baltic are a safe food source for human consumption because their BPA and alkylphenol contents are low.

Endocrine-disrupting compounds (EDCs) are defined as chemicals that impact on endocrine system structure or function. They could interface with the synthesis, secretion, transport, binding, action, and elimination of natural hormones in organisms that are responsible for the maintenance of homeostatic, reproduction (including embryonic development, gonadal formation, and sex differentiation), growth, digestion, behavior, adiposity, and cancer (Flint et al. 2012).

Endocrine activity has many widely studied organic compounds, such as polycyclic aromatic hydrocarbons, polychlorinated biphenyls, and organochlorine pesticides. A lesser-known group of compounds, in terms of occurrence and transfer in the food web of marine organisms, are derivatives of phenol. Mainly used in the production of surfactants and as additives in plastics, these include alkylphenols (NP, OP) and BPA.

Phenol derivatives can mimic the action of the sex hormone 17β-estradiol. There have been frequent reports of the feminisation or hermaphroditism of fish inhabiting areas close to waste outlets, and similar disorders can be observed in birds and mammals. In humans, BPA, OP, and NP disrupt development and lead to cancer of the sex organs: testicles, prostate, and breasts (Kang et al. 2006a, b; Markey et al. 2011).

The bioaccumulation of NP has been studied in various species of algae, plants, invertebrates, and freshwater fish and determined to be of a low or medium level (Ahel et al. 1993; Snyder et al. 2001). Little is known about the bioaccumulation of OP and BPA.

Previous studies performed on the content and transformations of alkylphenols have concentrated mainly on freshwater species and, for the most part, bioaccumulation was determined in laboratories. There are currently very few publications on the distribution and transportation of these compounds in the food web of animals living in the wild, and, so far, no such studies have been performed in the Baltic Sea.

The Gulf of Gdańsk, situated in the southern part of the Baltic Sea, is exposed to contaminants. The first studies, undertaken by Staniszewska and Falkowska (2011), indicated the presence of OP and NP (on average 3.0 [OP] and 34.5 ng dm^−3^ [NP]) in the surface water of the gulf’s coastal zone and pointed to the fact that these compounds occurred in considerable quantities in the microlayer of the water surface. With the exception of one-off measurements, no studies have previously been performed on the occurrence of BPA, OP, and NP in organisms of the coastal zone of the Southern Baltic.

The aim of the present study was to determine the concentration levels of BPA, OP, and NP in selected organisms of the marine food web, thereby making it possible to assess the bioaccumulation of these compounds. The studies included phytoplankton, zooplankton, mussels (*Mytilus edulis trossulus*), herring (*Clupea harengus*), cod (*Gadus morhua*), and flounder (*Platihthys flesus*), which constitute the major components of the food web in the Gulf of Gdańsk (Sokołowski 2009). Mussels are particularly good organisms for biomonitoring of the environment because they filter relatively large quantities of seawater but lack the ability to metabolise the pollutants and therefore tend to accumulate various toxic compounds present in the immediate environment. Their diet consists mainly of phytoplankton and suspended particles from the surrounding water (Wołowicz et al. 2006). Cod, herring, and flounder are all important from a human health perspective because, in the Baltic Sea, these are the most frequently caught fish species intended for human consumption.

The study also encompassed gulls (*Larus argentatus* and *L. marinus*), which occupy a high position in the food web. These birds mostly inhabit the coastal zone of the sea, where they feed on organisms from various links of the trophic chain, but they also consume food of anthropogenic origin (domestic refuse and waste dumped from fishing boats). They are thus widely regarded as excellent bioindicators of environmental pollution (Furnes and Camphuysen 1997).

## Materials and Methods

### Seawater, Phytoplankton, Zooplankton, Mussels, Fish, and Aquatic Birds

**Fig. 1:**
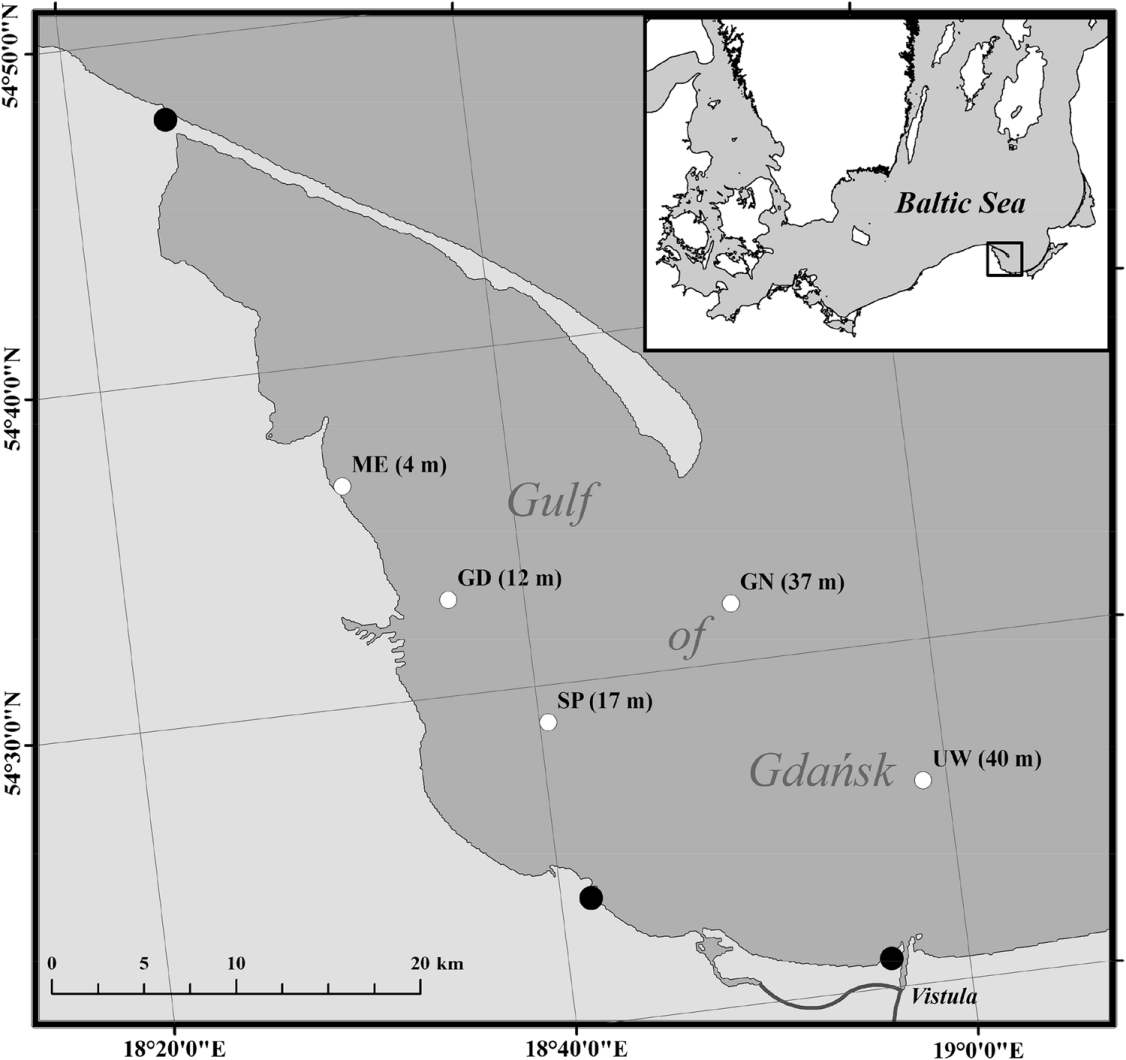
Location of sampling stations in the Gulf of Gdańsk. *Open circles* represent collection stations for the samples of water, phytoplankton, zooplankton, and mussels. *Closed circles* represent gull habitats

Samples of water, phytoplankton, zooplankton, and mussels were taken in the coastal zone of the Gulf of Gdańsk onboard r/v Oceania (IO PAN Sopot) between 18 and 20 March 2011. Test stations were located in water between 4 and 40 m deep close to potential pollution sources: the Dębogórze water purification plant (ME), Gdynia harbour (GD), Sopot (seaside resort), the outlets of several streams, including the Kacza River (SP), the Vistula River estuary (UW), and a GN station located at some distance from the coastal zone (Fig. 1).

Water samples (1 dm^3^) were taken onboard the ship into bottles made of dark glass using a special system (subsurface layer from a depth of 0.5 m]) and a bathometer (bottom water) and were kept at a temperature of 4 °C for no longer than 72 h.

Phytoplankton was collected using a WP2-type 25-µm mesh plankton collection net (surface pull lasting approximately 20 min) and sieved through a 100-µm mesh (to separate it from zooplankton and large suspended particles). Zooplankton was collected using a “Copenhagen” collection net with an inlet diameter of 50 cm and a 100 µm diameter mesh performing between 15 and 25 vertical pulls. In the laboratory, live zooplankton was separated from the dead and phytoplankton. Live zooplankton was placed in a darkened container, from where it was transported through a narrow pipe to a lit container filled with seawater devoid of organisms. After 90 min, the water from the lit container was sieved through a 100-µm mesh and, thus, live concentrated zooplankton was obtained. The phytoplankton and zooplankton collected for the determination of BPA, OP, and NP was then frozen and lyophilised.

Mussels (*M. edulis trossulus*) were collected with the use of a bottom dredge and kept for 24 h in aerated seawater of the same temperature and salinity as in the near-bottom water environment to remove the alimentary content. Then on the basis of the macroscopic and microscopic characteristics of the sex organs, sex was determined for those mussels measuring between 30 and 40 mm. Having separated the male and female specimens, the soft tissue was lyophilised, weighed, and homogenised in three pools containing seven individuals each. The weight index (CI) was calculated based on the proportion of each specimen’s body weight to its length (Beukema and de Bruin 1977) as follows:

1 $$\text{CI} = W \cdot L^{ - 3} \cdot 1000$$

where *W* = weight (g) and *L* = length (cm).

The sampled herring (*Clupea harengus*), cod (*Gadus morhua*), and flounder (*Platihthys flesus*) were caught in the years 2008–2011 from the Gulf of Gdańsk and obtained dead directly from the fishermen. All of the fish were weighed whole. Individual herring samples consisted of whole fish and collective samples of muscles and livers taken from 11 specimens. As with cod and flounder, muscle tissue and livers were extracted.

The biometric parameters of these mussels, herring, cod, and flounder, as well as their CI which were calculated using Eq. (1), are listed in Table 1. Dead birds were collected for sampling between 2010 and 2011 in Władysławowo, on the beach at Gdańsk Stogi, and at the mouth of the Vistula River (Fig. 1). In the laboratory during dissection, their sex was determined and their muscle tissue and livers extracted. The tests were performed on 11 birds age 2–5 years: 10 European herring gulls (5 males and 5 females) and 1 great black-backed gull. The age of each bird was determined based on plumage characteristics (Malling Olsen and Larsson 2004). Gull guano was collected on the breakwater at Tolkmicko in the Vistula Lagoon (Fig. 1) in November and December 2011. The biological material was always homogenised first, then lyophilised and homogenised again.

**Table 1:** Biometric data of mussels (*M. edulis*), herring (*C. harengus*), flounder (*P. flesus*), and cod (*G. morhua*) from the Gulf of Gdansk (caught between 2008 and 2011)

Species	*W* (g) minimum–maximum	Average *W* ± SD	*L* (cm) minimum–maximum	Average *W* ± SD	CI (mg cm^3^) minimum–maximum	Average CI ± SD
Mussels (M) (*n* = 5)	0.07–0.26^a^	0.16 ± 0.05^a^	3.2–4.0	3.6 ± 0.2	1.35–7.65	3.39 ± 1.34
Mussels (F) (*n* = 5)	0.09–0.29^a^	0.16 ± 0.05^a^	3.2–4.0	3.6 ± 0.3	0.21–5.81	3.48 ± 1.08
Herring (*n* = 10)	86–706	261 ± 188	–	–	–	–
Flounder (*n* = 6)	495–1,050	708 ± 236	33.0–44.0	38.0 ± 3.9	0.83–1.79	1.29 ± 0.35
Cod (*n* = 6)	560–1,400	928 ± 313	34.0–51.0	43.0 ± 6.7	1.01–1.53	1.14 ± 0.20

*W* weight, *L* length

^a^Dry weight of soft tissue

### Determination of BPA, OP, and NP and Method Validation

All solvents (water, acetonitrile, and methanol) were of high-performance liquid chromatography (HPLC) grade and were purchased from Merck. Seventy percent chloric acid (VII) and ammonium acetate (analytically pure) were purchased from POCh. High purity (>97 %) BPA, OP,4and NP were obtained from Sigma-Aldrich. Stock and working solutions (respectively: 1 mg cm^−3^ and 10, 25, 50, 75, 100 ng cm^−3^) of each compound were prepared in methanol.

To determine BPA, OP and NP, 0.5 g of the biological material, previously extracted in an ultrasonic bath, and 250 ml of water were filtered first through a 0.45-µm filter (CHROMAFILGF/PET-45/25). This was then purified on Oasis HLB (Waters) glass cartridges (5 ml/200 mg) according to the procedure set out by Xiao et al. (2006) for organisms and by Hou et al. (2006) for water with little-changing solvent volumes. The final determinations were performed using HPLC with fluorescence detection (Dionex; excitation at *λ* = 275 nm and emission at *λ* = 300 nm). Chromatographic separation was performed on a Hypersil GOLD reversed-phase column (Thermo Scientific) with a mobile phase (acetonitrile and water) under gradient conditions. All operations at the BPA, OP, and NP collection and determination stages were performed using tools and containers without plastic elements.

The linear correlation coefficient (*r*) of the analytical standards was >0.999. The average amounts of BPA, NP, and OP recovered, as determined through a quintuple analysis of samples containing a known amount of the standard, were 83.7 % (BPA), 87.4 % (OP), and 85.6 % (NP). The achieved accuracy (variation coefficient) was <15.0 % for each of the compounds. The limit of quantification (LOQ) was determined as a tenfold signal-to-noise ratio for a sample with a very low analyte content and amounted to 5.0 (BPA), 1.0 (OP), and 4.0 (NP) ng dm^−3^ for water and 2 (BPA), 0.8 (OP), and 1.0 (NP) ng g^−1^ dw for organisms. The obtained “background” values for BPA, NP, and OP were <LOQ. Only for BPA in water sample analyses did the “background” fall within the range of <LOQ to 6.5 ng dm^−3^ depending on the batch of solid phase extraction columns. Therefore, the background was checked every time a new batch of columns was used. The bioconcentration (BCF), bioaccumulation (BAF), and biomagnification (BMF) factors were determined according to the formulas presented by Hu et al. (2005).

The relationships between the concentrations of the studied compounds and the age, weight, and condition of a specimen (depending on the species) were established on the basis of correlation coefficients (*r*) with statistical significance of *p* ≤ 0.05 as confirmed using Spearman’s test.

## Results

BPA was detected in all water, phytoplankton, zooplankton, and mussel (*M. edulis trossulus*) samples from the Gulf of Gdańsk. OP was not detected in 20 % of samples and NP in only one sample (Table 2). The highest water-based concentration among the studied compounds was that of OP, which was 2–9 times greater than the OP concentration and approximately twice as high as BPA and on average amounted to 59.2 ng dm^−3^ in surface water and 79.2 ng dm^−3^ in bottom water. The highest OP concentrations in surface water were recorded at the UW station (the mouth of the Vistula River) and the SP station (near Sopot beach and the Kacza River), whereas the highest NP concentrations were noted at the ME station (close to a water purification plant) and the UW station. The highest NP concentrations in the bottom waters were recorded at the SP station near the Kacza River (Table 2). In contrast, BPA concentrations in surface water varied only slightly between the different stations. In the biota, however, the concentration of BPA was found to be the highest, with concentrations in phytoplankton, zooplankton, and mussels approximately 14 times greater than the OP concentration and approximately 5 times greater than the NP value. In plankton, BPA concentrations fell within the range of 30.5–769.2 ng g^−1^ dw, and in mussels the range was 6.8–197.2 ng g^−1^ dw (Table 2). The highest BPA concentrations were recorded in phytoplankton and zooplankton at the SP station, near the Kacza River, and NP and OP had the greatest concentrations in plankton at the UW station (the mouth of the Vistula River). However, in mussels the greatest BPA and NP concentrations were found at the SP station (near the Sopot beach and Kacza River), and the greatest OP concentrations were observed near the water purification plant at Dębogórze (ME) and Gdynia Harbour (GD) (Table 2).

**Table 2:** Concentrations of BPA, OP, and NP in samples of surface water, bottom water, plankton, and mussels (*M. edulis trossulus*) from the Gulf of Gdansk (April 2011)

Sampling station/variable	Subsurface layer (ng dm^−3^)			Bottom water (ng dm^−3^)			Phytoplankton (ng g^−1^ dw)			Zooplankton (ng g^−1^ dw)			Mussels (ng g^−1^ dw)		
	BPA	OP	NP	BPA	OP	NP	BPA	OP	NP	BPA	OP	NP	BPA	OP	NP
UW	34.0	65.9	77.3	25.5	43.1	103.6	53.0	87.5	140.6	105.7	27.1	263.7	–	–	–
SP	38.2	45.8	19.9	45.5	<LOQ	172.1	90.9	16.6	65.3	322.0	<LOQ	160.6	F: 29.5	F: 0.8	F: 66.1
													M: 122.2	M: <LOQ	M: 39.9
ME	44.6	<LOQ	132.9	10.3	<LOQ	4.1	239.2	25.1	159.7	769.2	<LOQ	4.5	F: 19.0	F: 2.9	F: 19.0
													M: 6.9	M: 1.1	M: 22.4
GN	29.6	5.8	12.9	67.7	<LOQ	51.2	74.5	55.1	20.8	325.4	3.9	16.3	–	–	–
GD	48.1	4.9	52.8	48.0	<LOQ	64.8	30.5	5.7	68.3	507.1	<LOQ	<LOQ	F: 23.0	F: 1.5	F: 30.0
													M: 28.8	M: 4.2	M: 38.8
*n*	5	5	5	5	5	5	5	5	5	5	5	5	10	10	10
Minimum	29.6	<LOQ	12.9	10.3	<LOQ	4.1	30.5	5.7	20.8	105.7	<LOQ	<LOQ	6.8	<LOQ	18.8
Maximum	48.1	65.9	132.9	67.7	43.1	172.1	239.2	87.5	159.7	769.2	27.1	263.7	197.2	6.4	75.6
Average	38.9	24.6	59.2	39.4	9.0	79.2	97.6	38.0	90.9	405.9	6.4	89.1	43.3	1.8	39.1
SD	7.6	29.5	48.7	22.1	19.1	63.0	82.3	33.2	57.6	247.9	11.7	118.2	56.7	1.9	20.6

*LOQ limit of quantification*, *n* number of samples, *SD* standard deviation

LOQ for organisms = 2.0 (BPA), 0.8 (OP), and 1.0 ng g^−1^ dw (NP); LOQ for water = 5.0 (BPA), (OP), and 4.0 ng dm^−3^ (NP)

BPA and NP were detected in all specimens of fish, i.e., herring (*C. harengus*), flounder (*P. flesus*), and cod (*G. morhua*) and gulls, *i.e.*, herring gulls (*L. argentatus*) and one black-backed gull (*L. marinus*) from the Gulf of Gdańsk. In fish, BPA and NP concentrations fell within the ranges of 19.7–798.4 and 5.4–41.9 ng g^−1^ dw, respectively. OP concentrations ranged between 1.0 and 89.2 ng g^−1^ dw, but some fish samples (12.5 %) showed concentration levels <LOQ (Table 3). In muscles and livers of gulls, BPA and NP were detected within the ranges of 6.2–317.7 and 5.0–76.6 ng g^−1^ dw, respectively. OP was not found in all of the tissues and organs of gulls, but, where it was, concentrations ranged between <0.8 (in 18 % samples) and 33.7 ng g^−1^ dw (Table 4). Particularly high concentrations of BPA (41.6–2701.9 ng g^−1^ dw) and NP (7.3–299.0 ng g^−1^ dw) were found in guano of herring gulls from the Gulf of Gdańsk, whereas the OP concentrations in guano were the lowest among the studied compounds, ranging between <0.8 and 16.9 ng g^−1^ dw (Table 4).

**Table 3:** BPA, OP, and NP (ng g^−1^ dw; **ng g^−1^ lw) in herring (*C. harengus*), flounder (*P. flesus*), and cod (*G. morhua*) from the Gulf of Gdansk (2009–2011)

	Herring									Flounder						Cod					
	Whole body			Muscle*			Liver*			Muscle			Liver			Muscle			Liver		
Compound	BPA	OP	NP	BPA	OP	NP	BPA	OP	NP	BPA	OP	NP	BPA	OP	NP	BPA	OP	NP	BPA	OP	NP
Minimum	19.7	2.5	5.4	–	–	–	–	–	–	98.3	1.0	11.8	55.1	<LOQ	11.5	25.4	<LOQ	6.3	87.1	5.7	8.3
Maximum	440.1	50.2	41.9	–	–	–	–	–	–	755.7	19.2	33.1	407.1	45.2	32.8	798.4	9.5	26.3	309.2	89.2	38.2
Average	105.8	19.0	24.0	98.6	<LOQ	9.8	81.0	1.1	5.2	430.4	10.2	18.6	250.5	16.1	18.8	236.3	3.0	11.6	164.0	27.7	22.2
Average**	11.3	2.0	2.6	10.6	<LOQ	1.0	–	–	–	7.7	0.2	0.3	–	–	–	0.7	0.01	0.03	–	–	–
SD	126.6	15.1	10.8	–	–	–	–	–	–	264.5	6.8	7.7	133.3	16.5	7.9	293.3	3.4	7.4	82.7	31.7	10.9
*n*	10	10	10	1(11)	1(11)	1(11)	1(11)	1(11)	1(11)	6	6	6	6	6	6	6	6	6	6	6	6

LOQ = 2.0 (BPA), 0.8 (OP), and 1.0 ng g^−1^ dw (NP)

* Livers and muscles tissues from 11 herrings

** Average results converted on lipid weight (ng g^−1^ lw): average contents of lipids in muscle tissues of herring = 10.7 g, of cod = 0.3 g, of flounder = 1.8 g in 100 g of tissue (Kunachowicz et al. 2005)

**Table 4:** Mean concentrations of BPA, OP, and NP (ng·g^−1^ dw) in tissues (and guano) of herring gulls (*L. argentatus*) and a black-backed gull (*L. marinus*)

	Muscles			Liver			Guano		
Herring gulls									
Variable	BPA	OP	NP	BPA	OP	NP	BPA	OP	NP
Minimum	6.3	<LOQ	5.0	6.2	<LOQ	5.1	41.6	<LOQ	7.3
Maximum	156.4	20.5	43.3	317.7	33.7	76.6	2701.9	16.9	299.0
Average	62.9	5.3	13.7	111.0	11.7	20.0	996.4	4.6	74.0
SD	52.1	6.9	11.1	116.6	9.2	20.7	1031.3	5.8	102.5
*n*	10	10	10	10	10	10	7	7	7
Black-backed gull (*n* = 1)									
	39.8	<LOQ	29.5	324.4	10.1	16.2	–	–	–

LOQ = 2.0 (BPA), 0.8 (OP), and 1.0 ng g^−1^ dw (NP)

## Discussion

### Factors Affecting Concentrations of BPA, OP, and NP in Water and Biota

The main factors affecting concentrations of BPA, OP, and NP in water, planktonic organisms, and mussels from The Gulf of Gdańsk are inland. Estuaries and stations around sewage-treatment plants were characteristic of stations where the greatest concentrations of the tested compounds were found (Table 2). In spring 2011 (after a long and snowy winter), OP and NP concentrations in surface water were probably greatly influenced by meltwater supplied by the Vistula River and smaller rivers such as the Kacza River. In earlier studies, Staniszewska and Falkowska (2011) indicated the high participation of the Kacza River (the largest of the many streams in the area of Sopot) in supplying OP to the surface water of the gulf. The Kacza River is also a source of the high concentration of NP in bottom waters at the SP station (Table 2). An additional factor in this may be that conditions at this station, located at a depth of 17 m, are probably conducive to the accumulation of detritus. Shallow stations, such as ME (Dębogórze water purification plant), where sediments contain little detritus, were characterised by lower NP concentrations compared with surface water. Large fluctuations in concentrations of the studied compounds in mussels were found to depend on the position of the station in relation to inland sources. Because no differences were observed in the physiological condition of mussels from different locations, it seems likely that they were collected during a prespawning period (Wolowicz et al. 2006). This seems to confirm that no correlation exists between BPA and alkylphenol concentrations, and biological parameters of mussels, such as length, weight, and condition.

The effect of habitat on BPA, OP, and NP concentrations was also observed with organisms higher on the trophic chain and because the habitats of fish and birds cannot be precisely characterised by geographical coordinates, this had to be performed through their diet. The highest concentrations of the analysed compounds occurred in muscle and livers of flounder and cod, and the lowest were found in herring (Table 3). Cod belong to the pelagic-benthic food web and feed mainly on smaller fish. Flounder are part of the benthic food chain, and young specimens feed mostly on zooplankton, whereas adults eat bivalves, snails, and insect larvae. Herring are fish of the pelagic food web and feed mostly on zooplankton, but adult specimens prefer juvenile fish (Terofal and Militz 1997). However, after recalculating the results according to lipid content, the greatest BPA, OP, and NP concentrations in muscle tissue were discovered in herring, because it the fattiest of the fish, followed by flounder and then cod (Table 3).

The level and duration of exposure to many contaminants from the EDC group are reflected in the concentrations of these compounds in the liver. The contaminants first pass through the liver, after which the compound reaches the muscles (Mita et al. 2011). Markey et al. (2011) showed that in rainbow trout (*Oncorhynchus mykiss*) and carp (*Cyprinus carpio*), NP was accumulated mainly in liver, fatty tissue, and kidneys. A liver/muscle concentration ratio of >1 testifies to prolonged exposure to endocrine compounds introduced to the system with food (Havelková et al. 2008; Kwaśniak et al. 2012). In the fish of the Southern Baltic, the lowest OP and NP concentrations in liver were found in the planctivorous herring, whereas the highest were found in the pescivorous cod. The mean relation between the liver/muscle concentrations of NP and OP for flounder and cod was >1 and for herring was <1.

The highest values of the liver/muscle OP ratio, 20–40, occurred in cod. It is conceivable that these predatory fish from the Baltic pelagic-benthic food web are the most exposed to this compound as a result of their diet. This seems to be confirmed by the high Spearman’s correlation coefficient (OP *r* = 0.9, NP *r* = 0.7; *n* = 6, *p* ≤ 0.05) determined between the liver/muscle concentration ratio and the weight of individual cod. The larger and older the fish, the greater the OP and NP concentrations in liver. Hence, liver is a good indicator of marine environmental pollution. Researchers pointed out the importance of liver in processes of storing, redistributing, detoxifying, and transforming contaminants depending on age and sex (Burger and Gochfeld 2007; Havelková et al. 2008; Kwaśniak and Falkowska 2012).

In the present study, BPA accumulation was greater in muscles than in livers of fish (liver/muscle concentration ratio was on average <1). This testifies to short-term exposure to this compound in the organism despite the fact that the observed BPA concentrations were much greater compared with those of OP and NP in both muscles and livers. It would also appear that the removal of BPA from the livers of flounder and herring, as well as older cod, occurs more rapidly. BPA has the highest polarity among all of the studied phenol derivatives (log K_o/w_ values vary between 2.2 and 3.4 [Staples et al. 1998]) and hence is more easily removed from the liver.

Several studies have shown a greater liver/muscle concentration ratio for BPA in laboratory-exposed fish compared with those collected from their natural habitat. However, in areas particularly affected by pollution for longer periods, BPA accumulation in liver has been noted to increase. Belfroid et al. (2002) observed BPA concentrations 15-fold greater in liver than in muscle of flounder at the mouth of the Erms River, whereas, in the unpolluted open waters of the Wadden Sea, the liver/muscle concentration ratio amounted to approximately 0.3–0.4.

In the top predators from the Gulf of Gdańsk, the black-backed gull (*L. marinus*) and the herring gulls (*L. argentatus*), a mean liver/muscle concentration ratio of >1 indicates prolonged alimentary exposure to BPA, OP, and NP (Kwaśniak et al. 2012). A particularly high liver/muscle concentration ratio was obtained for OP, reaching an average of 2.2–12.6. The diet of herring gulls in the coastal zone of the Gulf of Gdańsk is varied. BPA, OP, and NP concentrations (Table 5) found in muscles and livers of herring gulls wintering near the Gulf of Gdańsk (the fishing port area in Władysławowo) were 1–13 times greater compared with those in nonwintering gulls. In winter, food is more difficult to obtain and herring gulls, which reside close to fishing ports, supplement their diet, consisting mainly of anthropogenic waste, with fish snatched from fishing boats and with fish waste disposed of after the catch. Large black-backed gulls prefer natural food, mainly fish and animal waste as well as smaller gulls, but in winter, as with the omnivorous herring gulls, they look for food in landfill sites. OP and NP concentrations in muscles and livers of black-backed gull were low compared with those of the herring gulls, and this may be explained by the marine diet of the former species (Meissner et al. 2007). The greatest BPA concentration found in the liver of a herring gull was probably the result of a large proportion of food of anthropogenic origin, thus proving the importance of the gulls’ habitat and diet (Table 5). Increased concentrations of the measured compounds in wintering gulls compared with summer migrants could have been influenced by the seasonal fluctuation in air quality that occurs in winter, when fuel and waste combustion peaks. At this time, various toxic compounds are emitted into the air in greater volumes, including BPA, OP and NP, all of which have been identified in small PM1 and PM2.5 particles in the air of the Southern Baltic’s coastal zone (nonpublic data, Staniszewska et al.).

**Table 5:** Mean concentrations of BPA, OP, and NP (ng g^−1^ dw) in tissues of herring gulls (*L. argentatus*) with a distinction between wintering and nonwintering specimens, as well as between males and females, in the coastal zone of the Gulf of Gdańsk

Species	Muscle			Liver		
	BPA	OP	NP	BPA	OP	NP
W (*n* = 5)	74.9	8.4	16.3	119.3	14.8	22.2
NW (*n* = 5)	44.9	0.6	9.8	98.5	7.2	16.8
F (*n* = 5)	29.8	0.9	8.5	92.0	11.9	13.6
M (*n* = 5)	80.9	11.2	12.7	109.5	10.7	13.8

*W* wintering, *NW* nonwintering, *F* female, *M* males

An important parameter influencing the accumulation of BPA and alkylphenols may be the age of the tested organisms. Such dependence has been found in fish (Kwaśniak et al. 2012), although not in birds (Szumiło et al. 2013); therefore, the levels of BPA, OP, and NP determined in skeletal muscles and livers of fish may have been influenced by age-related changes to diet. A positive correlation between concentrations of BPA, OP, and NP in livers of fishes and whole herring, and the weight of fish, appears to support this (Table 6) observation, and similar relationships have been found in Baltic fish by, among others, Reindl et al. (2013) and Kwaśniak et al. (2012).

**Table 6:** Relationship between BPA, OP, and NP concentrations in tissues of flounder *(P. flesus),* cod (*G. morhua*), and whole herring (*C. harengus*) and the weight of fish from the Southern Baltic (2009–2011)

Fish	Tissue	BPA	OP	NP
Flounder (*n* = 6)	Muscles	0.7	0.6	0.7
	Liver	0.8	0.8	0.6
Cod *(n* = 6)	Muscles	0.7	−0.7	−0.6
	Liver	0.7	0.9	0.8
Herring (*n* = 10)	Whole body	1.0	0.7	0.5

*r* (Spearman’s correlation coefficient) *p* ≤ 0.05)

The negative correlation between OP and NP concentrations in muscles and the weight of cod (Table 6) was probably influenced by the lower fat content in cod compared with herring and flounder (Reindl et al. 2013; Kwaśniak and Falkowska 2012). The observed liver/muscle concentration ratio for NP and OP, which in cod increased with weight (age), could have resulted from the length of exposure to xenobiotics.

The most significant factors influencing the concentrations of BPA and alkylphenols are the sex of birds and mussels. This is confirmed by the fact that BPA, OP, and NP concentrations determined in gulls of the Gulf of Gdańsk were between 1 and 12 times greater in muscles and livers of males than in females (Table 5). Takeuchi et al. (2004) and Kim et al. (2003) indicated faster BPA metabolism in female than in male mammals (rats and humans). Furthermore, the speed of BPA removal increases in pregnant mammals (Kang et al. 2006a). Similar studies have not been performed on birds, but greater BPA, OP, and NP concentrations in males compared with females (Table 5) point toward processes similar to those discovered in mammals. Greater concentrations of the studied compounds observed in male specimens of mussels, BPA = 2.5-fold on average, for OP = 1.5-fold and for NP = 0.9-fold (Table 2), may imply the existence of different detoxication systems in mussels of different sexes.

### Bioconcentration, Bioaccumulation, and Biomagnification of Phenol Derivatives in the Food Web

BCF, BMF, and BAF factors determined for BPA, OP, and NP in organisms of the Gulf of Gdańsk that were >1 (United States Environmental Protection Agency 1991) signify the accumulative potential of the given compounds (Table 7). However, according to the recommendations from (United States Environmental Protection Agency 1991), as long as the factor does not exceed 100, the accumulation is not significant. The Stockholm Convention (2001) established that only when bioaccumulation factors exceed 5,000 can the substance be classified as undergoing dangerous biological accumulation. BCF values obtained in the present study for BPA, OP, and NP were relatively high averaging 0.1 to 10.9 × 10^3^, whereas BAF and BMF factors achieved lower values (maximum BAF and BMF values were between 20 and 30) and indicate the potential of these compounds to accumulate in the trophic chain of the Gulf of Gdańsk (Table 7).

**Table 7:** BCF, BAF, and BMF determined for BPA, OP, and NP in organisms of the Gulf of Gdansk (2008–2011)

Ratio	BPA		OP		NP	
	Average	Maximum	Average	Maximum	Average	Maximum
	BCF × 10^3^					
Phytoplankton/water (*n* = 5)	2.5	5.4	7.5	25.1	1.8	3.3
Zooplankton/water (*n* = 5)	10.1	17.2	0.2	0.7	2.6	8.1
Mussel/water (*n* = 10)	1.1	4.3	1.8	6.4	1.3	5.5
Herring/water (*n* = 10)	2.7	11.3	0.5	1.3	0.6	0.9
Flounder/water (*n* = 6)	10.9	17.2	1.1	2.1	0.2	0.4
Cod/water (*n* = 6)	6.1	20.5	0.1	0.4	0.2	0.4
	BAF					
Zooplankton/phytoplankto*n* (*n* = 5)	6	17	<1	<1	1	3
Mussel/phytoplankton (*n* = 10)	<1	3	<1	1	<1	1
Herring/phytoplankton (*n* = 10)	1	5	<1	1	<1	<1
Herring/zooplankton (*n* = 10)	<1	<1	<1	<1	<1	<1
Flounder/mussel (*n* = 6)	10	16	6	11	<1	1
Cod/mussel (*n* = 6)	6	18	2	5	<1	1
Cod/herring (*n* = 6)	2	8	<1	<1	1	2
Herring gulls/herring (*n* = 10)	1	2	<1	1	1	4
	BMF					
Herring gulls/herring (*n* = 10) (muscles)	1	2	5	21	2	4
Herring gulls/herring (*n* = 10) (liver)	2	4	11	31	4	15
Herring gulls/flounder (*n* = 10) (muscles)	<1	<1	1	2	1	2
Herring gulls/flounder (*n* = 10) (liver)	<1	1	1	2	1	4
Herring gulls/cod (*n* = 10) (muscles)	<1	1	2	7	1	2
Herring gulls/cod (*n* = 10) (liver)	1	2	<1	1	1	4

BCFs determined for BPA in phytoplankton, zooplankton, and mussels from the Gulf of Gdańsk tended to be several times greater than those for NP and OP (Table 7). The greatest of these BCF values was >17 × 10^3^ (average: 10 × 10^3^) and was determined for zooplankton. BCFs presented in literature are characterised by a wide range of values. For example, BCF values calculated for BPA in mussels vary from 1.7 to 4.1 (Ekelund et al. 1990; Ricciardi et al. 2008) and for NP from 1.5 to 26,000 (Granmo 1991). The results obtained from the Gulf of Gdańsk during this study proved bioaccumulation between phytoplankton and zooplankton and indicated zooplankton to be slightly richer in BPA than phytoplankton (BAF average = 6; BAF maximum = 17) during spring. Relatively low BAF values may reflect favorable conditions for the development of both phytoplankton and zooplankton. No further BPA bioaccumulation was observed in mussels (BAF < 1) from the Gulf of Gdańsk. With OP and NP, the bioaccumulation in these compounds had probably halted at the zooplankton level (BAF ~ 1) (Table 7). Correa-Reyes et al. (2007) showed that alkylphenols accumulation halted at a low trophic level\, observing that the crustacean *Artemia fransiscana*, feeding on the microalga *Isochrysis galbana* (previously incubated with NP), was able to metabolise and remove NP from its system very quickly, as a result of which the compound did not undergo bioconcentration. Despite these findings, however, there is also information on bioaccumulation at greater trophic levels (McLeese et al. 1981; Snyder et al. 2001), and the results of the present study confirm that slight accumulation of phenol derivatives occurred at a greater level of the trophic chain (Table 7).

The results from the Gulf of Gdańsk have shown that accumulation of phenol derivatives in fish, as expressed by the BCF factor, depends more on the environmental exposure of a particular species, and on the ability to metabolise, than on the position the species occupies in the trophic chain. A number of laboratory experiments have shown that bioaccumulation generally occurs only with high doses of BPA and that at low doses it is biodegraded or metabolised by the organism (Staples et al. 1998). High BPA, OP, and NP concentrations in food and in the environment can thus have a decisive influence on bioaccumulation. In Asian countries, where the observed concentrations of phenol derivatives in the environment are several orders of magnitude greater than those in Europe or North America (Wang et al. 2010), there are numerous reports on the significant bioaccumulation of these compounds. In countries where concentrations are considerably lower, however, for instance in Canada, OP and NP are not classified as compounds undergoing bioaccumulation (Klecka et al. 2005). Kang et al. (2006a) showed that numerous organisms at various trophic levels (from bacteria to mammals) have the ability to degrade and metabolise BPA. This depends mainly on the individual abilities of a given species, e.g., strains of freshwater bacteria can deal with BPA degradation much quicker (within a matter of a few days) than marine bacteria (>30 days). In contrast, McLeese et al. 1981) proved that the half-life of alkylphenols in mussels is much shorter than that in fish, which undoubtedly has an important effect on the difference in BCF, BAF, and BMF values in both organism groups. Snyder et al. (2001) reported a BAF approximately 100 times greater in fish (*fathead minnow*), 246–434 for NP, than in clams and mussels. In the present article, the maximum observed BAF concentrations for BPA and OP were only 5–18 times greater for flounder and cod than for mussels, whose BAF values for NP were close to 1 (Table 6).

Values of BAF factors for BPA, OP, and NP in fish occur in the literature in a wide range. For instance, Staples et al. (1998) reported BCFs for rainbow trout (*O. mykiss*) exposed to BPA within the range of 5–68 (laboratory); Snyder et al. (2001) indicated BAFs for NP in fathead minnow (*Pimephales promelas*) of 245–380 (laboratory); and Ahel et al. (1993) noted BAF values for OP and NP in fish from the Glatt River in Switzerland (*Squalius cephalus, Barbus barbus, O. mykiss*) between 13 and 410.

In the case of seabirds, the most important role in accumulation and magnification is probably played by diet. Its influence is showed more clearly in the latter (Table 7). Comparatively high BMF values (liver herring gull/liver herring), particularly for OP, can be explained by the gulls’ winter diet, which consists mainly of landfill waste supplemented with fish and fish guts discarded from fishing boats and its less efficient removal. Mechanisms concerned with the transformation and detoxification of phenol derivatives in birds have not been well researched. The few available findings suggest that BPA is accumulated in liver, from where it is quickly removed with bile and then expelled from the organism together with guano (quails and chickens) and that a small portion may penetrate into eggs (Halldin et al. 2001). Rapid BPA removal (within a couple of days) has been similarly observed in mammals (mice and rats) through faeces and urine (Markey et al. 2011).

In the present study, the considerable role of guano in the removal of phenol derivatives is very noticeable. High BPA and NP concentrations in guano testify to birds’ ability to detoxify in this way (Table 4). This seems to be confirmed by the fact that BPA and NP concentrations in gull guano were 3.1 (NP) and 9.4 (BPA) times greater than in the herring, which they would have habitually consumed. However, fish comprise only part of the gulls’ diet in the coastal area of the Gulf of Gdańsk, and additional doses of BPA and NP originate from landfill sites, especially during the winter when herring gulls (*L. Argentatus*) flock to such areas in the thousands to feed (Meissner et al. 2007).

The gulls’ ability to eliminate BPA and NP from their organism and the supremacy of this process over accumulation are also suggested by the fact that concentrations of these compounds were found to be greater by at least 5.4 for NP and 15.8 for BPA in guano than in various tissues. In contrast, the supremacy of accumulation over detoxication is indicated by low OP concentrations in guano compared with the food (herring) and tissues of the gulls (ratio 0.2–0.9).

Constant consumption of food contaminated with BPA and NP was probably responsible for the significant relationship between BPA and NP concentrations in liver of the birds and their age (BPA *r* = 0.7, *p* < 0.05; NP *r* = 0.6, *p* < 0.05). With OP, there was no statistically significant correlation.

BPA and NP present in guano penetrate back into the environment, particularly in and around breeding and residing sites, which are often situated at beaches and breakwaters and where seabirds can be found *en masse*. Many investigators, among them Blais et al. (2005), have stressed the significant role of migrating birds in the transportation of toxic mercury and pesticides in guano over short and long distances. This is an important contributing factor in the occurrence of BPA, OP, and NP away from potential sources of emissions as in the North Sea (Ebindhaus and Xie 2006).

### Risk of BPA, OP, and NP to the Environment of the Gulf of Gdańsk

In general, concentrations of phenol derivatives in water, mussels, fish, and birds from the Gulf of Gdańsk are comparable with or lower than those in other European regions and are much lower than those in Asia (Blackburn et al. 1999; Jonkers et al. 2003; Wang et al. 2010). In seawater, concentrations of alkylphenols and BPA range from several hundred pg dm^−3^ (nonpolluted areas) to several µg dm^−3^ (polluted areas). Particularly high alkylphenol concentrations were characteristic of the coastal waters and estuaries of England, (0.1–2.6 µg dm^−3^) Denmark (0.031–0.934 µg dm^−3^), and Spain (0.4–4.1 µg dm^−3^) (Blackburn et al. 1999; Jonkers et al. 2003). Lower values comparable with the results of the present study were found in the North Sea with concentrations of NP and OP along the German coast measuring between 2.5 and 13.8 and between 0.11 and 0.6 ng dm^−3^, respectively (Beck et al. 2006) and BPA concentration in the coastal waters of Denmark returning average values of 14–40 ng dm^−3^ (Belfroid et al. 2002). In addition, Lilja et al. (2009), in one water sample from the Gulf of Gdańsk (near Gdynia), determined respective NP, OP, and BPA concentrations of 63, <0.5, and <14 ng dm^−3^, respectively, whereas a previous study by Staniszewska and Falkowska (2011) in the same area noted NP and OP concentrations of 34.5 and 3.0 ng dm^−3^, respectively.

Concentrations of BPA, OP, and NP in organisms greater than those obtained in the present study were determined by Wang et al. (2010) on the northeast coast of China, where the presence of NP in mussels was recorded at a level of 44–7,600 ng g^−1^ dw, and by Hu et al. (2005), who detected NP at a level of 99.4–372.7 ng g^−1^ dw in fish of the Bohai Gulf, also in the northeast of China. More comparable concentrations of <2–2,380, <1.5–7.4, and <1–1,479 ng g^−1^ wet weight (ww) were found for BPA, OP, and NP, respectively, in *Mytilus edulis* caught off the coasts of Denmark and Norway (Hansen & Lassen 2008). In addition, in whole fish caught off the coast of Denmark, the presence of the studied compounds was determined at the following levels: BPA <1–233, OP < 1.5, and NP 75–6,925 ng g^−1^ ww. In the small collection of data for Baltic fish, published by Helsinki Commission (2010), one can find results similar to those in the present article. In muscles of herring, perch, and flounder (caught off the coasts of Estonia, Latvia, Lithuania, Poland, and southeastern Sweden), the determined concentrations were BPA < 0.6–3.9, OP < 1, and NP < 0.6–23 ng g^−1^ ww (Lilja et al. 2009; HELCOM 2010).

There is no information concerning BPA, OP, and NP content and distribution in tissues and organs of birds from the Baltic Sea region. However, concentrations of NP in muscles of herring gulls from the Bohai Gulf (139.7–372.7 ng g^−1^ dw) (Hu et al. 2005), and in muscles and liver of mallard ducks (*Anas boscas*) from the Glatt River in Switzerland (Ahel et al. 1993), are greater by two orders of magnitude than those found in birds from the Gulf of Gdańsk.

The environmental concentration/predicted no effect concentration (EN/PNEC) ratio was devised to evaluate the risk of BPA, OP, and NP present in the environment, with PNECs in water set at 150, 10, and 330 ng dm^−3^ for BPA, OP, and NP respectively (Helsinki Commission 2010; European Union Risk Assessment Report 2010). The calculated EC/PNEC ratio for the maximum concentrations of each compound from the Gulf of Gdańsk reached 0.45 (BPA), 6.6 (OP), and 0.52 (NP) and were <1. Under this classification scheme, and based on the present concentrations for BPA and NP in surface and bottom water, ecotoxicological effects are not likely to occur in the Gulf of Gdańsk. For OP in the coastal zone near Sopot (SP) and the mouth of the Vistula River (UW station), the EC/PNEC ratio was >1; thus, ecotoxicological effects may be observed but only occasionally.

### Estimation of Human Exposure to the Ingestion of Phenol Derivatives Present in the Fish from the Southern Baltic

In many articles, it has been suggested that fish and seafood ingestion is one of the main sources of human exposure to phenol derivatives. This is confirmed by the relationship discovered between the amount of fish and seafood consumed and concentrations of BPA, OP, and NP found in human tissues, serum, and milk (Lopez-Espinosa et al. 2009; Mita et al. 2011; Ferrara et al. 2011). Ferrara et al. (2011) estimate that the average daily intake of phenol derivatives is approximately 20 times greater in people with high seafood consumption. Moreover, as humans come into contact with plastic or waste combustion fumes in their everyday lives, they are constantly exposed to BPA that penetrates into their digestive or respiratory system (Kang et al. 2006b).

The average consumption of fish and fish products in Poland is estimated at approximately 35.0 g day^−1^ (World Trade Organization 2011). The tolerable daily intake (TDI) set for BPA by European Food Safety Authority (2007) is 50 µg kg^−1^ body weight day^−1^, whereas the TDI for NP set by the Danish Environmental Agency is 5 µg kg^−1^ body weight day^−1^ (Lopez-Espinosa et al. 2009). A TDI has not been established for OP, but a no observed adverse effect level (NOAEL) of 10 mg kg^−1^ body weight day^−1^ was reported for rats by Tyl et al. (1999).

Concentrations of BPA, OP, and NP determined in muscles of herring, flounder, and cod caught in the Gulf of Gdańsk are not high enough to pose a threat to humans. Assuming that consumption consists entirely of muscles, and taking into account the maximum concentrations found, one daily BPA dose for a human weighing 70 kg would amount to a mere (based on the wet weight of fishes) 0.11 µg day^−1^, whereas in the case of OP and NP it would be only 0.006 µg day^−1^, respectively. These doses are lower than the allowed TDI limits (in the case of OP, the dose is much lower than the NOAEL). These daily doses are also approximately 10–20 times lower than those indicated in other countries, e.g., Germany, Italy, New Zealand, and Taiwan (Ferrara et al. 2011).

## Conclusion

The concentration results obtained for BPA, OP, and NP in the coastal area of the Gulf of Gdańsk (the Baltic Sea) represent the first such set of data for the ecosystem of the Gulf of Gdańsk and indicate that this region is not contaminated with BPA, OP, or NP and is therefore, in this regard, rather safe for humans. BPA and NP concentrations found in water do not pose an ecological threat, but ecotoxicological effects may occasionally be observed for OP (EC/PNEC ratio >1). In living organisms, concentrations of BPA, OP, and NP ranged between LOQ and several hundred ng g^−1^ dw. BPA, OP, and NP concentrations determined in muscles of herring, flounder, and cod caught in the Gulf of Gdańsk, introduced to the organism by way of its food, do not pose a threat to the health of humans.

In most cases, long-term alimentary exposure to BPA, OP, and NP (liver/muscle concentration ratio >1) was observed for both fish and birds. Thus, accumulation of BPA, OP, and NP in fishes and gulls from the Gulf of Gdańsk was probably associated mainly with habitat and diet. The highest concentrations of EDCs related to the lipid content were found in muscles and livers of the pelagic herring and the lowest in predatory flounder and cod.

The diet of herring gulls, consisting mostly of waste from landfill sites and supplemented with fish from harbours and fishing wharfs, could have influenced the greater BPA, OP and NP concentrations found in muscles and livers of gulls wintering in the vicinity of the Gulf of Gdańsk compared with nonwintering birds. The sex of birds and mussels is the most significant factor influencing the concentrations of BPA and alkylphenols, and BPA, OP, and NP concentrations were found to be greater in male specimens than in females for both the gulls and mussels of the Gulf of Gdańsk.

Maximum BAF values calculated for BPA, OP, and NP, ranging between 20 and 30, confirms the possible bioaccumulation of these compounds in the food web, particularly on lower levels of the trophic chain (zooplankton and mussels).

It can be concluded that the process of EDC removal has supremacy over the process of accumulation in tissues and organs of birds. This seems to be confirmed by high concentrations determined for BPA (≤2,700 ng g^−1^ dw) and NP (≤300 ng g^−1^ dw) in guano of herring gulls compared with concentrations found in fish. Guano, deposited in close vicinity to the sea, becomes a secondary source of EDCs in the environment.
